# A Systematic In Silico Mining of the Mechanistic Implications and Therapeutic Potentials of Estrogen Receptor (ER)-α in Breast Cancer

**DOI:** 10.1371/journal.pone.0091894

**Published:** 2014-03-10

**Authors:** Xin Li, Rong Sun, Wanpeng Chen, Bangmin Lu, Xiaoyu Li, Zijie Wang, Jinku Bao

**Affiliations:** School of Life Sciences and Key Laboratory of Bio-Resources and Eco-Environment, Ministry of Education, Sichuan University, Chengdu, China; University of Wisconsin - Madison, United States of America

## Abstract

Estrogen receptor (ER)-α has long been a potential target in ER-α-positive breast cancer therapeutics. In this study, we integrated ER-α-related bioinformatic data at different levels to systematically explore the mechanistic and therapeutic implications of ER-α. Firstly, we identified ER-α-interacting proteins and target genes of ER-α-regulating microRNAs (miRNAs), and analyzed their functional gene ontology (GO) annotations of those ER-α-associated proteins. In addition, we predicted ten consensus miRNAs that could target ER-α, and screened candidate traditional Chinese medicine (TCM) compounds that might hit diverse conformations of ER-α ligand binding domain (LBD). These findings may help to uncover the mechanistic implications of ER-α in breast cancer at a systematic level, and provide clues of miRNAs- and small molecule modulators- based strategies for future ER-α-positive breast cancer therapeutics.

## Introduction

Breast cancer is the most common cancer in women in industrialized countries [1]. Estrogen receptor (ER)-α activation appears in approximately 70% of breast cancer and plays a critical role in the pathogenesis of ER-α-positive breast cancer [2], making it not only an important prognostic marker and clinical outcome indicator, but also a potential therapeutic target in breast cancer.

Selective estrogen receptor modulators (SERMs) and selective estrogen down-regulators (SERDs) have long been ideal choices for breast cancer treatment [3]. SERMs can competitively bind with ERs and then act as ER agonists or antagonists, while SERDs can modulate the turnover of ER in cells and tissues. Although several trials of SERMs have been launched over the past quarter century [4], the clinical outcomes may not be that satisfying. Hitherto, only tamoxifen (a first-generation SERM) and raloxifene (a second-generation SERM) have been approved by the Food and Drug Administration (FDA) for breast cancer treatment in the United States [5]. Other SERMs, such as the third-generation SERMs lasofoxifene, arzoxifene and bazedoxifene, are still in test. Of note, these drugs are mainly developed for osteoporosis treatment with breast cancer treatment as a secondary endpoint [6], which makes it in urgent need to develop novel ER-α modulators.

Intriguingly, microRNAs (miRNAs), small non-coding RNAs of ∼24 nucleotides (nt) in length [7], have been recorded to exhibit aberrant expression patterns and participate in the pathogenesis and endocrine resistance of multiple ER-α-positive breast cancer [8], [9]. Actually, there exist putative binding sites for several miRNAs in ER-α mRNA, some downstream miRNAs are regulated by ER-α, while other upstream miRNAs can regulate ER-α [10]. These miRNAs are not only potential diagnosis and prognosis markers, but also promising therapeutic targets in breast cancer [11]. Therefore, identifying target genes of ER-α-regulating miRNAs will help to explore the role of ER-α-related miRNAs, whereas predicting novel miRNAs targeting ER-α may promote the development of miRNA-based therapies.

In the past decades, advances in biological data collection and novel techniques have given rise to vast quantities of bioinformatic data in breast cancer research. However, these cancer data from different sources are always isolated and not exploited to their full potential [12]. This is surprising given that cancer is the outcome of global perturbations of cellular and molecular interactions rather than the disturbances of individual components [13], and the crosstalk of different cancer-associated modules in a biological system is important for effective drug discovery [14]. In the present study, we effectively integrated ER-α-related bioinformatic data from different sources at different levels, and identified novel miRNAs and traditional Chinese medicine (TCM) compounds that might target ER-α. Our findings might help to gain insight into the underlying mechanisms of ER-α at a systematic level, and thus help to maximize the potential of ER-α for future ER-α-targeted cancer therapies.

## Materials and Methods

### Collection of human ER-α-interacting proteins

With ER-α as the bait protein, we collected experimentally supported human ER-α-protein interaction pairs from five online protein interaction databases, including the Human Protein Reference Database (HPRD) (Release 9) [15], Database of Interacting Proteins (DIP) (July 2004 release) [16], IntAct (Release 165) [17], HomoMINT [18] (September 2001 release) and Biological General Repository for Interaction (BioGRID) (Version 3.2.99) [19] (detailed information of these five databases was given in Table S1). After removing duplicate interactions, the union of all interaction pairs was integrated into the human binary ER-α-protein interaction (EPI) network. Cytoscape (Version 2.8.2) [20] was used to visualize and present the EPI network.

### Retrieval of validated and predicted target genes of ER-α-regulating miRNAs

To explore the implications of miRNAs in ER-α signaling pathway, we searched literature available in Google Scholar [21] to obtain known miRNAs regulated by ER-α. Validated target genes of ER-α-regulating miRNAs were retrieved from the union of five experimentally verified miRNA-target interaction (MTI) resources, including the miRTarBase (Version 4.5) [22], TarBase (Version 6.0) [23], starBase (Version 1.0) [24], miRecords (Version 3) [25] and miRWalk (Last updated on March 29, 2011) [26] (detailed information of these databases was given in Table S2). Predicted target genes were acquired from the intersection of three algorithmically different programs, namely, DIANA-microT-CDS (Version 5.0) [27], miRanda (August 2010 release) [28] and TargetScan (TargetScanHuman Version 6.2) [29] (detailed information of the algorithmical difference and data filtering criteria was given in Table S3).

### Identification of indicated genes in MCF-7 cells via microarray analysis

To modify the target genes of ER-α-regulating miRNAs into a breast cancer-specific context, two transcriptional microarray datasets of human MCF-7 breast cancer cells (No. E-MTAB-1196 [30] and No. E-GEOD-10061 [31]) were downloaded from ArrayExpress database at the EMBL-European Bioinformatics Institute (EBI) (http://www.ebi.ac.uk/arrayexpress/experiments). The preliminarily experimental data were firstly log_2_ transformed and then normalized. Next, all data were organized into the “Two Class (unpaired)” response format in Significance Analysis of Microarrays (SAM) statistical method [32]. Subsequently, the fold change parameter was fixed to 2.0 to ensure genes change at least 2.0 fold compared with control group, and delta value in SAM plot controller was properly adjusted to limit the false discovery rate (FDR) within 5%. Then, gene expression signals were analyzed by T-statistic, and all identified significantly differential genes were used for further analysis.

### Analysis of functional GO annotations of indicated genes

Gene ontology (GO) annotations analysis was carried out to explore the biological meanings of ER-α-associated proteins. Using the GO project [33], we firstly searched GO functional terms, including cellular component (CC), molecular function (MF) and biological process (BP), against ER-α-interacting proteins and target genes of ER-α-regulating miRNAs. Subsequently, functional annotation clustering was performed *via* Database for Annotation, Visualization and Integrated Discovery (DAVID) (v6.7) [34], [35] to analyze the GO terms enrichment. Next, two standards, P value<0.05 and fold enrichment ≥2.0 were adopted to refine the GO terms set in major clusters. Then, Web Gene Ontology Annotation Plot (WEGO) [36] was used to integrate and plot the GO annotation analysis results.

### Prediction of miRNAs targeting ER-α

Since MTI prediction programs with different algorithms may lead to quite varying results, consensus predictions from at least two programs may help to predict reliable MTIs [37]. Herein, we combinationally adopted three algorithmically different programs, namely, DIANA-microT-CDS (Version 5.0) [27], miRanda (August 2010 release) [28] and TargetScan (TargetScanHuman Version 6.2) [29]. DIANA-microT-CDS is the only algorithm available online which identifies miRNA recognition elements (MREs) both in 3′ untranslated region (3′-UTR) and coding sequences (CDS), and the probability of being a real prediction is calculated as the miTG score [27]. We used a miTG score greater than 0.7 (a strictly high precision threshold) as the selection criterion. MiRanda uses miRanda algorithm to predict potential miRNA and mirSVR algorithm to rank the downregulation likelihood of miRNA with a mirSVR score [28]. Only predictions with “good” mirSVR score of −0.1 or lower were collected as reliable miRNAs. TargetScan predicts MTIs mainly based on the conservation by searching for conserved 8 mer and 7 mer sites that match the seed region of each miRNA [29]. MiRNAs with a context+ score of −1.67 or higher, appropriate *P*_CT_ value and at least one conserved site were selected as reliable predictions. Consensus predictions of these three programs were integrated as ER-α-targeted miRNAs.

### Validation of the MTI prediction model

To validate our MTI prediction model, we constructed a test dataset containing known false MTIs and true MTIs targeting four miRNAs hsa-miR-30a, hsa-miR-1, hsa-miR-155, and hsa-let-7b. For the false MTI dataset, proteomic data of protein expression changes while individually over-expressing those four miRNAs in HeLa cells were downloaded from the pSILAC database [38] (http://psilac.mdc-berlin.de), and only target genes having a logarithmic fold change exceeding 0 were chosen. Thereby, we obtained a false MTI dataset including 1475 MTIs for hsa-miR-30a, 1683 MTIs for hsa-miR-1, 1536 MTIs for hsa-miR-155, and 1458 MTIs for hsa-let-7b. For the true MTI dataset, experimentally supported human MTIs for the four miRNAs were downloaded from miRTarBase (version 4.5) (http://mirtarbase.mbc.nctu.edu.tw), and only functional MTIs with direct experimental evidence such as reporter gene assay were selected. Thus, we achieved a true MTI dataset containing 19 MTIs for hsa-miR-30a, 40 MTIs for hsa-miR-1, 58 MTIs for hsa-miR-155, and 15 MTIs for hsa-let-7b.

With the same selection criteria as used in our MTIs prediction (see previous descriptions), those four miRNAs were individually subjected to DIANA-microT-CDS, miRanda and TargetScan to predict potential MTIs, and the intersection results of those three programs were taken as candidate MTIs, namely “positive” predictions. Accordingly, true MTIs appeared in the intersection results were “true positive (TP)”, the others were “false negative (FN)”; false MTIs did not appear in the intersection results were “true negative (TN)”, the others were “false positive (FP)”.

Then, to assess the predictive performance of our model, accuracy (ACC), positive predictive value (PPV) and false-positive rate (FPR) were measured by the quantity of TP, TN, FP and FN as the formulas given below. According to the definitions and formulas, ACC measures the proportions of candidates that are correctly predicted, both true positives and true negatives, and it can assess how correct our model in detecting true MTIs and excluding false MTIs. PPV describes the percentages of positive results from a model that are true positive results, and it can estimate the probability a MTI will be true MTI when it appears in our prediction results. FPR depicts the proportions of negative candidates that are incorrectly predicted as “positive”, and it can evaluate the probability a false MTI will appear in our prediction results.
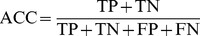
(1)

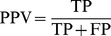
(2)

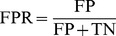
(3)


To confirm the superiority of our model than other strategies, we compared the predictive performance of individual program, combination of two programs, and integration of three programs.

### Virtual screening of TCM database for candidate ER-α modulators

#### Data preparation

The X-ray crystal structure of ER-α LBD in complex with 4-hydroxytamoxifen was downloaded from Protein Data Bank (PDB) (http://www.pdb.org/) (PDB entry: 3ERT) [39]. The ready-to-dock 3D structures of 27418 TCM compounds were downloaded from ZINC database (http://zinc.docking.org/catalogs/tcmnp) [40], updated at 2013-02-21.

Test dataset used for optimization and validation of our screening model was composed of 39 known ER-α binders (actives) and 1448 ER-α non-binders (decoys) from the Directory of Useful Decoys (DUD) Release 2 [41]. The 39 actives were taken from literature, and each active has about 37 decoys which share similar physical properties, such as molecular weight, cLogP, and number of hydrogen bonding groups, but dissimilar topological structure to its active counterpart.

#### Molecular docking procedure

UCSF DOCK6.3 program [42] with AMBER force field parameters was used to perform molecular docking screening. Proteins were prepared by UCSF Chimera (Version 1.8) [43], where solvent molecules were removed, hydrogens and standard charges were assigned to each receptor atom. Grid scoring was firstly used to rank candidate compounds with all the default parameters, and the best scoring pose of each compound towards the receptor was then subjected to amber scoring for re-ranking. PDB2PQR server [44] with AMBER force field was used to automate the PDB file preparation and protonation state assignments of ER-α, and the maximum number of orientations was set to 500.

To evaluate the performance of our two-phases docking procedure, test dataset was docked against the crystal structure of ER-α LBD, and then the receiver operating characteristic (ROC) curve and area under the ROC curve (AUC) were calculated with pROC package [45] in R software environment. The further away the ROC curve is above the diagonal and the closer the AUC value approaching 1, the better the docking performance. AUC was calculated as given below, where N_actives_ and N_decoys_ were the numbers of actives and decoys, and N^i^_decoys_1_ was the number of decoys ranked higher than the actives.
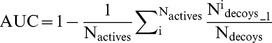
(4)


### Generation of ER-α LBD conformers

Considering that effective modulators should be able to hit diverse statuses of the target protein, we performed molecular dynamics (MD) simulations using GROMACS package (Version 4.5) [46] to generate a series of ER-α LBD conformers. Crystal structure of ER-α-OHT complex was used as the starting structure for MD simulations, and protein topology was processed by pdb2gmx with GROMOS96 43a1 force field [47]. Then, the system was equilibrated by two phase's equilibrations with the temperature and pressure maintained at 300 K and 1 bar for 100 ps, respectively. Finally, the 5000 ps MD simulations with a time step of 2 fs were launched at constant temperature (300 K) and pressure (1 bar). The resulted trajectory files were viewed and analyzed with VMD software [48], and all structural analysis were conducted using programs in GROMACS package.

To explore the conformational dynamics of ER-α LBD over the MD simulations, we monitored the variation of Cα atom root mean square deviation (RMSD), internal hydrogen bonds, secondary structure elements, and the per-residue root mean square fluctuation (RMSF). To accommodate as many conformational states of the protein as possible, snapshots of ER-α LBD with an interval of 200 ps were extracted, and thus resulted in an ensemble of 26 representative ER-α conformers.

### Screening of TCM compounds against valid ER-α LBD conformers

The pre-generated 26 ER-α LBD conformers were docked against the test dataset, and ACC, PPV and FPR of each conformer at different cut-offs were computed to evaluate their screening performance. And, only conformers exhibiting better performance than the crystal structure at specific cut-off were considered to be valid for the screening of TCM compounds. Then, those valid conformers were docked against 27418 TCM compounds with the two-phases docking procedure, and compounds ranking top 10% for all the valid conformers were considered as candidate ER-α modulators.

### Validation of the ER-α modulator screening model

The test dataset containing 39 known ER-α binders and 1448 non-binders was docked against each ER-α LBD conformer with the same docking procedure and parameters as used in our docking screening, and compounds that could hit all valid conformers were taken as candidate ER-α modulators, namely “positive” predictions. According to the formula (1) (2) (3), ACC, PPV and FPR of our screening model were calculated by the quantity of TP, TN, FP and FN, which denote true ER-α binders, true ER-α non-binders, false ER-α binders and false ER-α non-binders, respectively. Herein, ACC measures how correct our model in clarifying true ER-α binders and ER-α non-binders, PPV evaluates the probability a compound will be ER-α binder when it appears in our screening results, and FPR estimates the possibility an ER-α non-binder will appear in our screening results. To ensure the superiority of our screening model, we compared the performance of other strategies by taking compounds that could hit one or more valid ER-α LBD conformers as candidate ER-α modulators.

## Results

### Retrieval and functional GO annotation analysis of ER-α-interacting proteins

**Figure 1:**
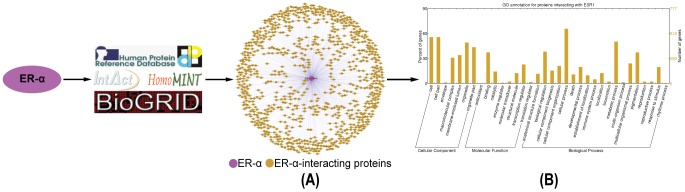
Retrieval and functional GO annotation analysis of human ER-α-interacting proteins. **A.** Human binary ER-α-protein interaction network. **B**. Functional GO annotation analysis of ER-α-interacting proteins.

Using ER-α as the bait protein, 732 protein pairs from BioGRID, 342 protein pairs from IntAct, 192 protein pairs from HPRD, 37 protein pairs from HomoMINT and 10 protein pairs from DIP were collected. All these interaction data are with certain experimental evidence. After removing duplicate interactions, we achieved a human binary EPI network containing 864 unique ER-α-protein interaction pairs (Figure 1A, Table S4). By requiring P value<0.05 and enrichment fold ≥2.0, DAVID clustered those 864 proteins into 116 functional clusters with 509 GO terms, including 80 CC terms, 71 MF terms and 358 BP terms. As shown in Figure 1B, WEGO integrated those functional clusters into 34 sub-categories with more than half of the proteins belonging to the GO functional groups cell (481 proteins, 55.7%; CC), cell part (481 proteins, 55.7%; CC), cellular process (568 proteins, 65.7%; BP) and metabolic process (434 proteins, 50.2%; BP).

### Functional GO annotation analysis of the target genes of ER-α-regulating miRNAs

**Figure 2:**
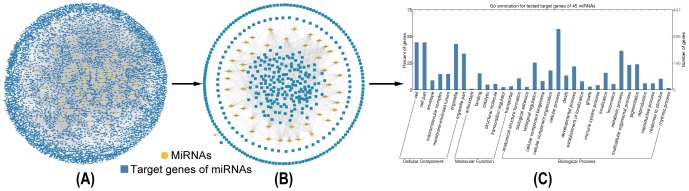
Identification and functional GO annotation analysis of the validated target genes of ER-α-regulating miRNAs. **A.** Global validated ER-α-regulating miRNA-target gene network. **B.** Validated ER-α-associated MTIs in MCF-7 cells. **C.** Functional GO annotation analysis of the validated target genes of ER-α-regulating miRNAs.

**Figure 3:**
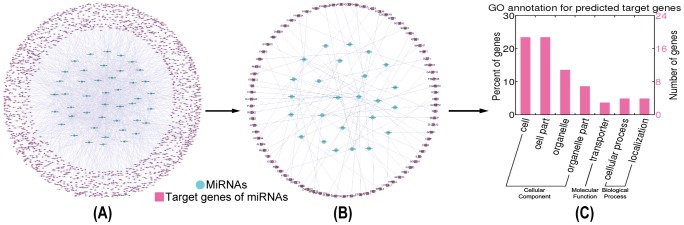
Identification and functional GO annotation analysis of the predicted target genes of ER-α-regulating miRNAs. **A.** Novel predicted ER-α-regulating miRNA-target gene network. **B.** Novel predicted ER-α-associated MTIs in MCF-7 cells. **C.** Functional GO annotation analysis of novel predicted target genes of ER-α-regulating miRNAs.

So far, 45 miRNAs (including 41 single miRNAs and 1 miRNA cluster) have been reported to be regulated by ER-α (Table S5). From the union of five platforms providing experimentally verified MTIs, namely, miRTarBase, TarBase 6.0, starBase, miRecords and miRWalk, we retrieved 7099 validated target genes of ER-α-regulating miRNAs (Figure 2A, Table S6). Additionally, predicted target genes of those 45 miRNAs were acquired from three algorithmically different programs DIANA-microT-CDS, miRanda and TargetScan, and their intersection resulted in 4163 unique predictions. Since these prediction programs rely highly on the validated MTIs [49], known target genes constituted a proportion of the prediction results overall. After deleting known MTIs, we finally obtained 1709 predicted target genes of ER-α-regulating miRNAs (Figure 3A, Table S7).

Subsequently, two transcriptional microarray datasets were utilized to integrate these target genes into a breast cancer-specific context. Based on the SAM analysis results, 1580 significantly differential genes were identified from untreated and drug-treated MCF-7 cells (No. E-MTAB-1196, Table S8), and 219 genes were identified from untreated and ER-α knockdown MCF-7 cells (E-GEOD-10061, Table S9). Then, previously obtained target genes were examined to see if they appeared in the differential gene set identified from the microarray data, and those not appeared were excluded. Consequently, we confirmed 596 validated and 81 predicted target genes of ER-α-regulating miRNAs in the context of MCF-7 cells (Figure 2B and Figure 3B, Table S10 and Table S11).

Afterwards, functional GO annotation enrichment analysis was performed against the target genes of ER-α-regulating miRNAs. As shown in Figure 2C, 596 validated target genes were classified into 33 sub-categories with more than 30% genes belonging to the GO functional groups cell (265 proteins, 44.5%; CC), cell part (265 proteins, 44.5%; CC), organelle (256 proteins, 43%; CC), organelle part (202 proteins, 33.9%; CC), cellular process (340 proteins, 57%; BP) and metabolic process (218 proteins, 33.6%; BP). And, 81 predicted target genes were sorted into 7 sub-categories with more than 20% genes belonging to the GO functional groups cell (19 proteins, 23.5%; CC) and cell part (19 proteins, 23.5%; CC) (Figure 3C).

### Prediction of novel miRNAs targeting ER-α

**Figure 4:**
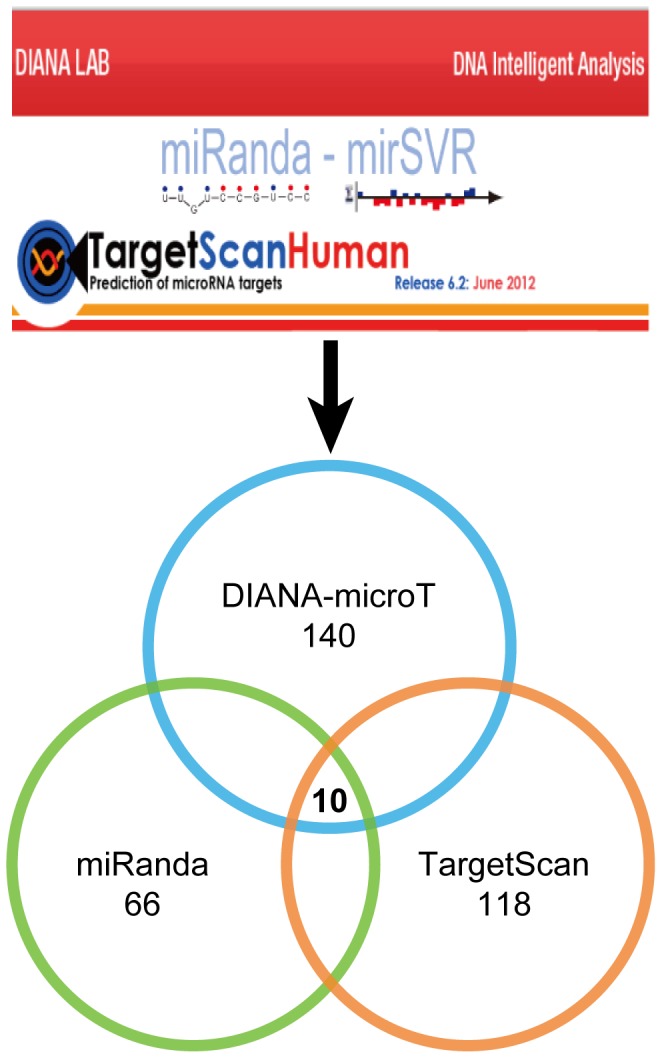
Prediction of novel miRNAs targeting ER-α from three algorithmically different programs.

To predict novel miRNAs targeting ER-α, we adopted three algorithmically different programs, including DIANA-microT-CDS, miRanda and TargetScan. These programs predict miRNAs mainly based on sequence complementarity between mature miRNA and target site, binding energy of miRNA-target duplex, and evolutionary conservation of target site sequence and target position in aligned UTRs [50]. Herein, with previously described data selection criteria, we predicted 140 miRNAs from DIANA-microT-CDS, 66 miRNAs from miRanda, and 118 miRNAs from TargetScan. Given high dependence of these prediction programs on known MTIs [49], known miRNAs constituted a proportion of the prediction results. After deleting known miRNAs, intersection of these three programs resulted in ten novel miRNAs, including hsa-miR-148b, hsa-miR-301b, hsa-miR-302e, hsa-miR-520a-3p, hsa-miR-520b, hsa-miR-520c-3p, hsa-miR-520d-3p, hsa-miR-520e, hsa-miR-874 and hsa-miR-1297 (Figure 4). Evaluation details of these novel miRNAs in each prediction program were given in Table S12. These miRNAs might inhibit the expression levels of ER-α and thus negatively regulating ER-α-mediated signaling pathways in breast cancer.

### Validation of the MTI prediction model

**Figure 5:**
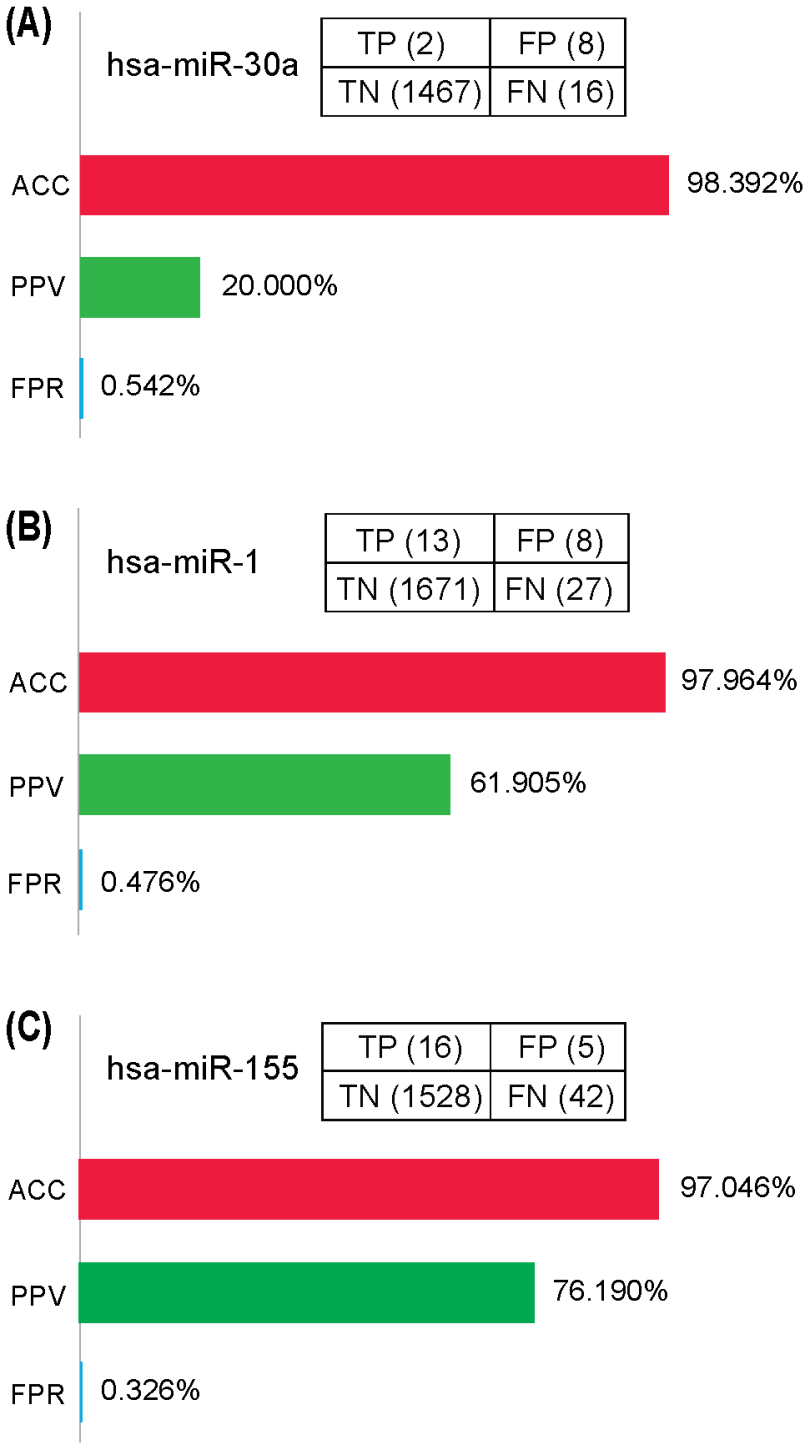
Validation of the MTI prediction model.

To validate our MTI prediction model, we utilized the test dataset containing known “false MTIs” and “true MTIs” targeting four miRNAs hsa-miR-30a, hsa-miR-1, hsa-miR-155, and hsa-let-7b. With the same data selection criteria as used in our MTI prediction, we predicted 285 MTIs for hsa-miR-30a, 192 MTIs for hsa-miR-1, 225 MTIs for hsa-miR-155, and 4 MTIs for hsa-let-7b. Then, we counted the number of TP, FP, TN and FN, and calculated the ACC, PPV and FPR of our prediction model (Figure 5, Table S13). Compared with the predictive performance of individual program, combination of two programs and integration of three programs, our model of selecting the intersection of three prediction programs displayed its superiority by showing the highest ACC, PPV, and the lowest FPR for all the four test datasets (Table S13). As for hsa-let-7b, although the intersection of three programs led to no positive predictions (TP+FP = 0), our model achieved the highest ACC (98.982%) compared with other strategies (Table S13). For other three datasets, our model exhibited the best performance with the ACC higher than 97%, PPV higher than 20%, and FPR less than 0.542% (Figure 5). These findings indicated that our model might be effective in detecting true MTIs and excluding false MTIs.

### Virtual screening of TCM compounds for candidate ER-α modulators

Given the inherent flexibility of protein structure, compounds that can hit variable conformations of the target protein may be able to modulate the interaction between protein and ligand at different stages, and thus have higher opportunity to be developed as potential modulators for clinical uses. Herein, we firstly explored the conformational dynamics of ER-α LBD over the 5000 ps MD simulations. Conformational elements of ER-α LBD, including the RMSD of Cα atoms, internal hydrogen bonds, secondary structures, and per-residue RMSFs, exhibited an irregularly time-dependent response during the whole simulation process (Figure S1). To accommodate as many conformational states of the protein as possible, we extracted conformation at 0 ps (the crystal structure) and 25 simulation-generated snapshots with an interval of 200 ps, and thus led to an ensemble of 26 representative ER-α LBD conformers.

**Figure 6:**
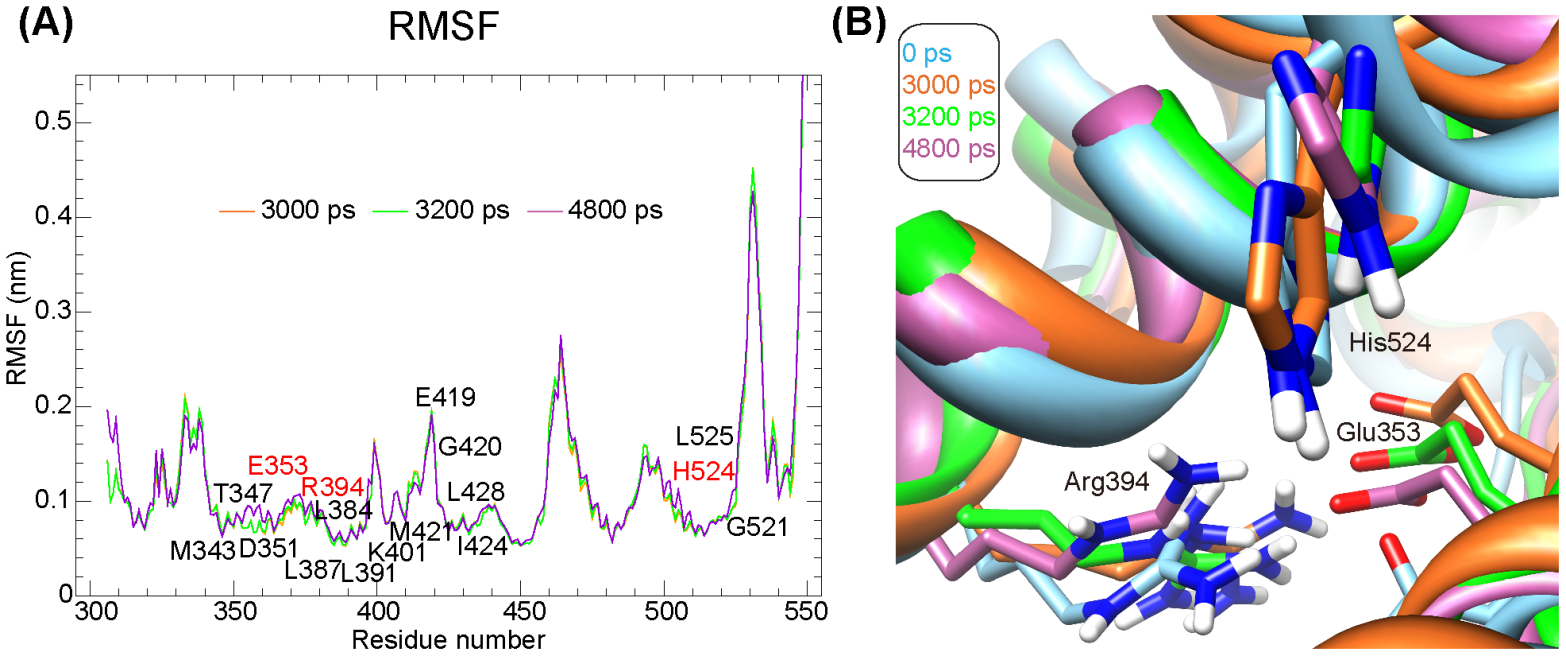
Conformational comparison of the four valid ER-α LBD conformers. **A.** Root mean square fluctuation (RMSF) analysis of three MD simulations-generated conformers with respect to the crystal structure of ER-α LBD. **B.** Pair-wise comparison of the catalytic triad (residues Glu353, Arg394 and His524).

To select conformers qualified for the screening of TCM compounds, we checked the screening performance of those 26 ER-α LBD conformers. After docking the test dataset into each conformer, we calculated their screening ACC, PPV and FPR at different cut-offs. Herein, we set ten cut-offs from 10% to 100%, which denotes that the top low-scored compounds at specific percentage were considered as candidate ER-α modulators, and then explored the optimal performance of each conformer at specific cut-offs. As for the crystal structure (conformation at 0 ps), a cut-off of 10% exhibited the best screening performance with ACC of 89.4%, PPV of 5.512%, and FPR of 8.602% (Table S14). As shown in Table S15, among other 25 conformers, three exhibited higher ACC, PPV, and lower FPR than crystal structure at the cut-off of 10%, namely, conformations at 3000 ps, 3200 ps and 4800 ps. Compared with the crystal structure (0 ps), RMSFs of key residues in the active sites of other three conformers displayed variable fluctuations ranging from 0.5∼2 Å (as labeled in the Figure 6A). And, cartoon of the pair-wise comparison of catalytic triad (residues Glu353, Arg394 and His524) that documented to be essential for the hydrogen bond interaction between E2 and ER-α was shown in Figure 6B. Together with the crystal structure, those four conformers were selected for further docking screening.

Then, 27418 TCM compounds were docked against each valid ER-α LBD conformer to screen potential ER-α modulators. As a result, we identified eighty compounds that could target all the four valid conformers, and their detailed screening information was given in Table S16. These compounds might have high potentials to serve as candidate ER-α modulators for future treatment of ER-α-positive breast cancer.

### Validation of the ER-α modulator screening model

**Figure 7:**
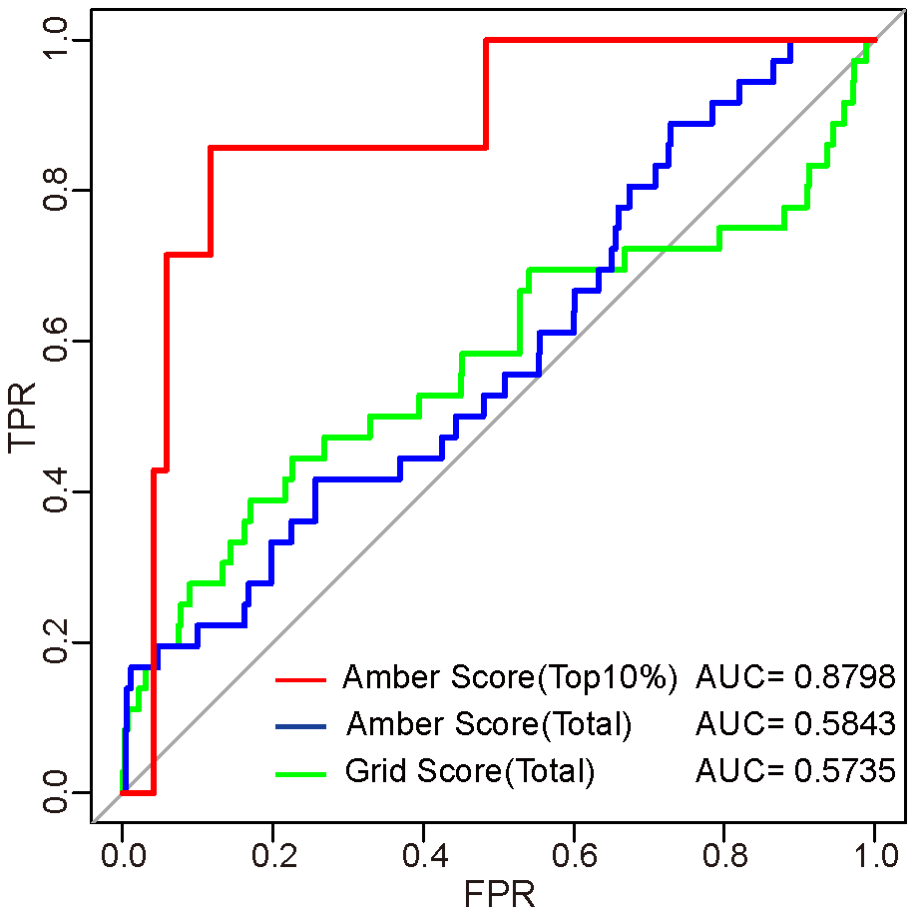
Comparison of the performance of three docking screening procedures.

To validate our docking screening model, test dataset containing 39 known ER-α binders and 1448 non-binders from the DUD (Release 2) was adopted. With the same procedure and parameters as used in our docking screening, we firstly evaluated the two-phases screening procedure by docking test dataset into the crystal structure of ER-α LBD. As shown in Figure 7, AUC value of grid scoring for total compounds was 0.5735, meaning a relatively slight discrimination capability. Moreover, amber scoring for all the grid-scored compounds led to an AUC value of 0.5843, suggesting an improved screening performance. While taking the top 10% amber-scored compounds as candidate ER-α modulators produced the highest AUC value up to 0.8798, indicating that our two-phases docking screening procedure of selecting top 10% amber-scored candidates from grid-ranked compounds could yield fairly acceptable screening performance.

**Figure 8:**
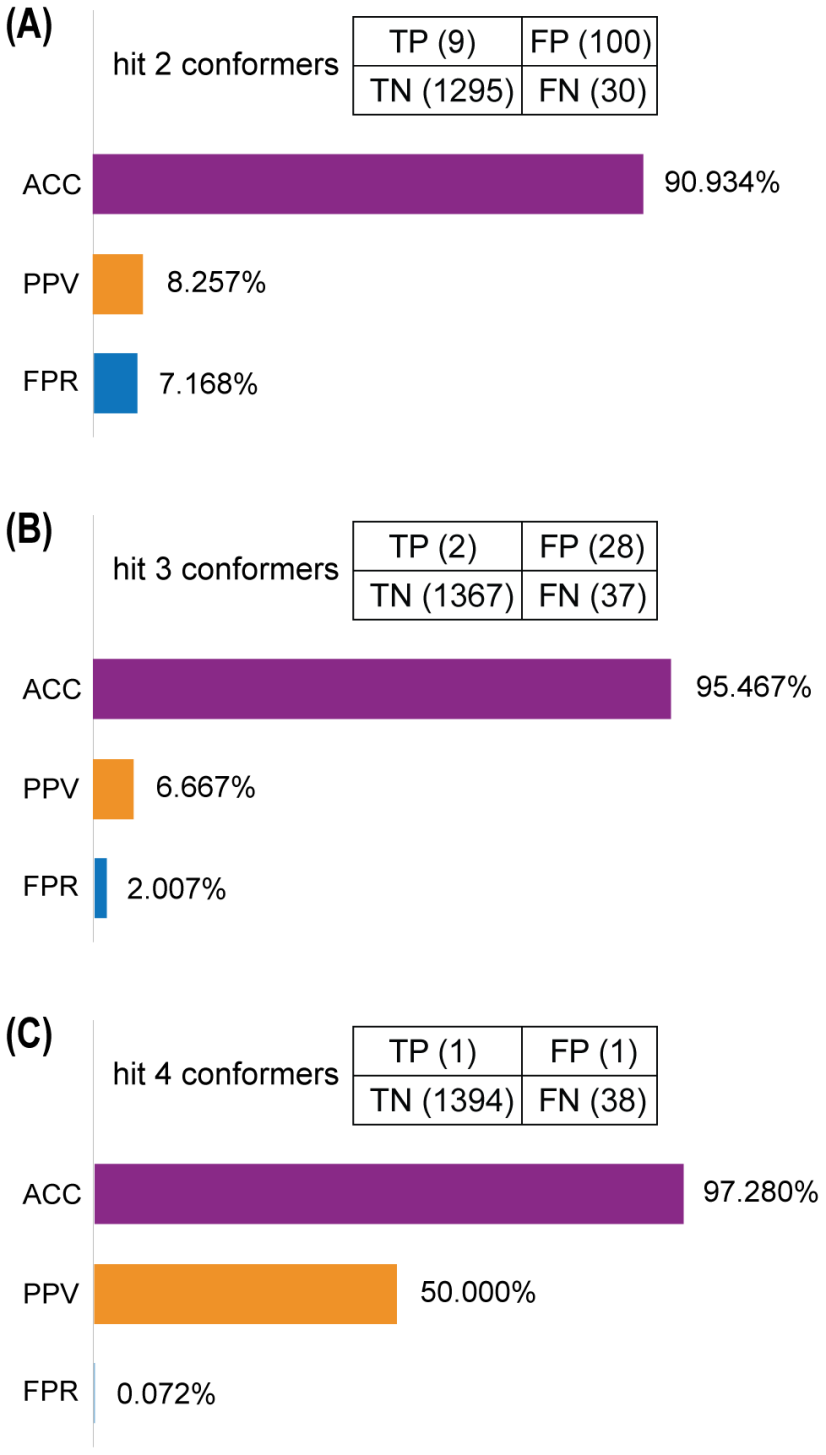
Validation of the ER-α modulator screening model. Comparison of the screening model by taking candidate modulators as those hitting two (**A**), three (**B**), or four (**C**) valid ER-α LBD conformers.

Then, we compared the performance of other strategies by taking compounds that could hit two, three, or four valid ER-α LBD conformers as candidate ER-α modulators. Compared with the screening performance of individual conformer (Table S15), using two or three conformers produced an enhanced performance with the ACC of 90.934% and 95.467%, PPV of 8.257% and 6.667%, and FPR of 7.168% and 2.007%, respectively (Figure 8A and B). However, using all the four valid conformers substantially improved the screening performance with the ACC of 97.280%, PPV of 50%, and FPR of 0.072% (Figure 8C). These results suggested that our model was able to screen potential ER-α modulators with the accuracy up to 97.280% and false-positive rate low as 0.072%, and candidate compounds identified to hit all the four valid conformers from our model had 50% chance to be true ER-α modulators (Table S16).

## Discussion

One of the notable characteristics of cancer is the appearance of protein sets that tend to physically interact with one another and work in cooperation [51]. Attempts have long been launched to identify potential interaction partners of known cancer-associated genes [52], [53]. And, various publicly available protein interaction databases [54], such as BioGRID, IntAct, HPRD, HomoMINT and DIP used in our study (Figure 1), have substantially facilitate the exploration of physical or functional associations among genetic components. Herein, we obtained 864 unique ER-α-protein interaction pairs (Figure 1A) with experimental evidence such as the yeast two-hybrid, mass spectrometry, peptide/protein array, immunohistochemistry, western blot, coimmunoprecipitation and fluorescence microscopy [15]. Further GO annotation analysis revealed that these proteins mainly located at cell and cell part, envelope, organelles and membrane-enclosed lumen, and participated in cellular process, metabolic process and biological regulation, *etc* (Figure 1B). Studies have confirmed the localization of ER-α in nucleus or non-nuclear subcellular fractions, including mitochondria and plasma membrane in MCF-7 cells [55], [56], and its discrepant ultrastructural distributions are closely associated with the action mechanisms of estrogen. In the nucleus, ER-α can function by binding directly to the estrogen response element (ERE) of target genes, or by interacting with transcription factors and recruiting of co-regulator proteins [57]. When redistributed to mitochondria, it can bind to mitochondrial EREs (mtEREs) in human mitochondrial DNA (mtDNA) [58], and thus regulating mtDNA gene transcription and modulating biological process such as mitochondrial respiration, glutathione distribution and apoptosis [59]. Moreover, plasma membrane-associated ER-α can act through the estrogen non-genomic signaling pathways *via* crosstalk with membrane receptors [60] and further stimulate downstream effectors [61]. Collectively, different subcellular localizations of ER-α and its associated proteins may exert diverse influence on their molecular function and biological process, and thus modulate corresponding physiologic and pathologic processes in breast cancer.

Aberrant expression patterns of miRNAs have been documented in different human breast cancer types [8], suggesting a potential role in breast cancer initiation and progression. Multiple techniques have been developed to identify miRNAs regulated by estrogen or ER-α, including global genome binding assays [62], miRNA microarrays [63] and luciferase reporter assays [64]. What's more, several resources are accessible to obtain experimentally verified target genes of indicated miRNAs, such as miRTarBase, TarBase 6.0, starBase, miRecords and miRWalk used in our work. And, some other programs are available to predict specific MTIs, such as DIANA-microT-CDS, miRanda and TargetScan adopted in our study. GO annotation analysis (Figure 1B, Figure 2C and 3C) suggested that both ER-α-interacting proteins and target genes (both validated and predicted) of ER-α-regulating miRNAs localized with the following order of priority: cell, cell part, organelle, and then organelle part. Moreover, ER-α-interacting proteins can perform a function of binding, catalytic, and transcriptional regulation, while only the target genes of ER-α-regulating miRNAs can function as transporters. In addition, all these ER-α-associated proteins were involved in the biological processes of cellular process and localization, and validated ER-α-related proteins could also participate in metabolic process, pigmentation, biological regulation, and developmental process, *etc*. These findings suggested that there existed certain identity and complementarity in cellular location and function between these two sets of ER-α-associated proteins.

Apart from being well known as critical regulators of cellular processes, miRNAs are also recognized as potential therapeutic targets in a wide range of diseases including cancer [7]. Given the significant implications of ER-α-related miRNAs, it is essential to predict novel miRNAs targeting ER-α for breast cancer therapeutic use. Although MTIs prediction programs with different algorithms may result in varying prediction results, studies have shown that at least a certain class of conserved MTIs can be confidently predicted [37]. In our case, we combinationally utilized three algorithmically different programs, and predicted ten novel miRNAs targeting ER-α (Figure 4, Table S12). With a test dataset containing known “false MTI” and “true MTI”, our prediction model was validated to have fairly acceptable predictive performance (Figure 5). Currently, several miRNA-based drugs, including anti-miRNAs and miRNA mimics, have been in clinical trials for the treatment of human diseases. For example, miravirsen, a locked nucleic acid (LNA)-based miRNA-122 inhibitor, is in Phase II clinical trial for patients with chronic hepatitis C virus (HCV) infection [65] (NCT01200420). MRX34, a miRNA-34a mimic compound, is now in the patient recruitment status for phase I study (NCT01829971), and is likely to be the first miRNA replacement compound reaching clinical trials [7]. Given the established therapeutic implications of miRNAs and the encouraging progress of miRNA-based therapeutic strategies, miRNAs predicted to target ER-α in our study might be potential for future treatment of ER-α-positive breast cancer.

In addition to miRNAs-based approaches, small molecule ER-α modulators may serve as an alternative therapy. Although existing ER-α-targeted SERMs, such as tamoxifen, raloxifene, ICI 164,384 and ICI 182,780, have achieved evidenced therapeutic effects, the situation that SERMs are now mainly developed for osteoporosis treatment and have not yet been widely approved for breast cancer treatment [5] prompts researchers and clinicians to explore novel ER-α modulators. As a complement to conventional wet lab high-throughput screening (HTS) methods, structure-based virtual screening approaches, including receptor- and ligand-based strategies, take advantage of the direct physical interaction between target proteins and ligand to identify novel lead scaffolds for further hit-to-lead optimization [66]. Recently, progresses have been achieved in the identification of novel ER-α modulators *via* structure-based virtual screening techniques. For example, eleven compounds have been screened as non-steroidal ER modulators from an in-house natural product database (NPD) containing over 4,000 natural products [67], and four novel ER-α antagonists have been obtained from SPECS library containing 160,796 commercial compounds *via* a structure-based *tieredScreen* protocol [68].

However, these existing *in silico* structure-based screening cases have not yet given enough consideration to the inherent flexibility of ER-α [69], which is an indispensable factor affecting the binding between ligand and target protein. Nowadays, experimental data for protein flexibility *in vivo* have been slow in coming due to costs and limitations in experimental techniques, therefore, MD simulations tend to be a good choice to model the conformational dynamics of target protein. Actually, the strategy of using an ensemble of protein conformations generated from MD simulations for subsequent docking screening as adopted in our study has been successfully confirmed in several reports. The earliest example of such routine was reported in 1994, in which Pang and colleagues obtained 69 conformations of acetylcholinesterase from the 40 ps MD simulations, and then identified huperzine A as its potent inhibitor *via* docking studies [70]. Several recent studies also confirmed the advantage of such screening procedure. For example, when adopting two-phases docking procedure to screen novel inhibitors of Avian influenza neuraminidase, researchers found that the “dynamic” model using representative receptor ensembles extracted from the 40 ns MD simulations could achieve better screening performance than the “static” model using single crystal structure of the protein [71]. Hitherto, such flexible screening strategy has not yet been applied to the screening of ER-α modulators. In this study, we combined MD simulations and molecular docking, where MD simulations were applied to generate a series of ER-α LBD conformers and molecular docking was utilized to evaluate the binding affinity between ER-α and candidate compounds. Using a test dataset containing known ER-α binders and non-binders, our screening model was validated to have relatively high predictive accuracy, positive predictive value, and low false-positive rate (Figure 8). The candidate TCM compounds (Table S16) identified from our two-phases screening endeavor may provide new cues in better harnessing TCM resources for future ER-α-targeted breast cancer therapeutics.

### Conclusions

In the present study, to better exploit the therapeutic potential of ER-α, we effectively integrated ER-α-related bioinformatic data from different resources at different levels. As a result, we identified human ER-α-interacting proteins and target genes of ER-α-regulating miRNAs, and predicted novel miRNAs and candidate TCM compounds that might serve as ER-α modulators. These inspiring findings may not only help to systematically illustrate the mechanistic implications of ER-α, but also provide new clues for future miRNAs- and SERMs-based therapies in ER-α-positive breast cancer.

## Supporting Information

Figure S1 **Conformational dynamics of ER-α LBD during the 5000 ps MD simulations.** **A.** The root mean square deviation (RMSD) of Cα atom. **B.** The stability of internal hydrogen bonds. **C.** The convergence of secondary structure elements. **D.** The per-residue root mean square fluctuation (RMSF) of Cα atom.
(TIF)

Table S1 **Overview of five online protein interaction databases used in our study.**
(PDF)

Table S2 **Overview of the resources providing experimentally verified MTIs in our study.**
(PDF)

Table S3 **Overview of the three algorithmically different programs providing predicted MTIs in our study.**
(PDF)

Table S4 **Unique ER-α-protein interaction pairs in the human binary EPI network.**
(XLS)

Table S5 **Reported ER-α-regulating miRNAs.**
(PDF)

Table S6 **Validated target genes of ER-α-regulating miRNAs.**
(XLS)

Table S7 **Novel predicted target genes of ER-α-regulating miRNAs.**
(XLS)

Table S8 **Significantly differential genes identified from transcriptional microarray data (No. E-MTAB-1196).**
(XLS)

Table S9 **Significantly differential genes identified from transcriptional microarray data (No. E-GEOD-10061).**
(XLS)

Table S10 **Validated target genes of ER-α-regulating miRNAs confirmed in MCF-7 breast cancer context.**
(XLS)

Table S11 **Novel predicted target genes of ER-α-regulating miRNAs confirmed in MCF-7 breast cancer context.**
(XLS)

Table S12 **Novel miRNAs predicted to target ER-α from three algorithmically different programs.**
(PDF)

Table S13 **Performance of different miRNA-target interactions (MTIs) prediction strategies targeting four test datasets.**
(PDF)

Table S14 **Screening performance of the crystal structure of ER-α LBD (conformation at 0 ps) at different cut-offs.**
(PDF)

Table S15 **Optimal screening performance of the 26 ER-α LBD conformers at specific cut-offs.**
(PDF)

Table S16 **Candidate ER-α modulators that might hit all the four valid ER-α LBD conformers.**
(PDF)
