# Supplementary figures and images for: A Systematic In Silico Mining of the Mechanistic Implications and Therapeutic Potentials of Estrogen Receptor (ER)-α in Breast Cancer
Source: PLoS One. 2014 Mar 10;9(3):e91894. doi: 10.1371/journal.pone.0091894 (PMC3948898; doi:10.1371/journal.pone.0091894)

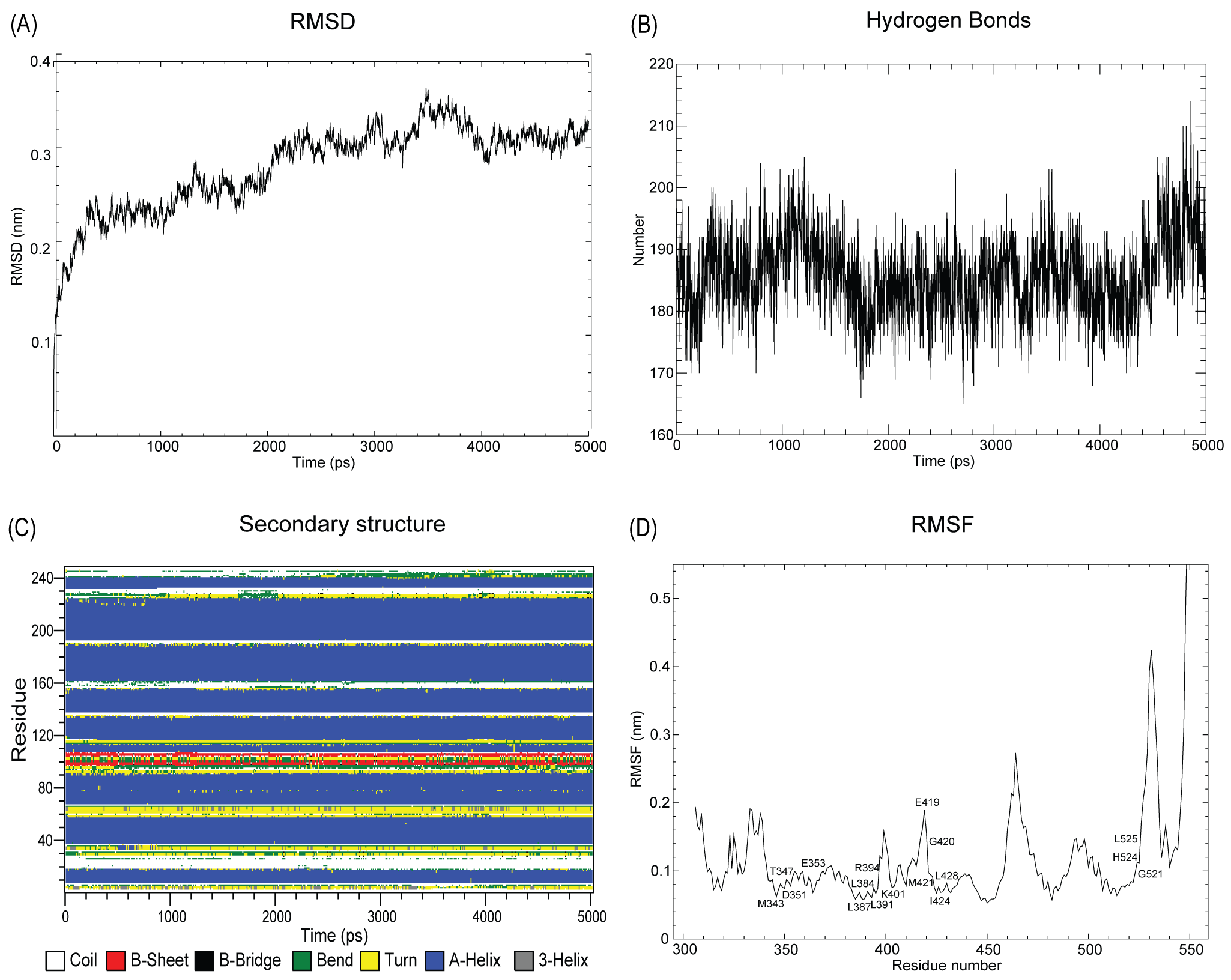

Supplement: Figure S1 — Conformational dynamics of ER-α LBD during the 5000 ps MD simulations. A. The root mean square deviation (RMSD) of Cα atom. B. The stability of internal hydrogen bonds. C. The convergence of secondary structure elements. D. The per-residue root mean square fluctuation (RMSF) of Cα atom. (TIF) [file pone.0091894.s001.tif]
