# Supplementary material for: A Systematic In Silico Mining of the Mechanistic Implications and Therapeutic Potentials of Estrogen Receptor (ER)-α in Breast Cancer
Source: PLoS One. 2014 Mar 10;9(3):e91894. doi: 10.1371/journal.pone.0091894 (PMC3948898; doi:10.1371/journal.pone.0091894)
Supplement: Table S1 — Overview of five online protein interaction databases used in our study. (PDF) [file pone.0091894.s002.pdf]

Table S1. Overview of the five online protein interaction databases used in our study.

Programs	Version information	Brief description	Experimental techniques	Web server	References
HPRD	Release 9, released on April 13, 2010	A database containing human protein-protein interaction data manually extracted from public literature and curated by expert biologists.	Yeast two-hybrid, mass spectrometry, immunohistochemistry, coimmunoprecipitation, peptide/protein array, fluorescence microscopy, and western blot.	http://www.hprd.org/	[15]
DIP	July 2004 release, updated on Jan 31, 2013	This data catalogs protein-protein interactions with experimental evidence curated manually by expert curators and also automatically using computational approaches.	High-throughput technologies such as large-scale yeast two-hybrid screens, protein microarray, and mass spectrometric analysis of affinity purified protein complexes.	http://dip.doe-mbi.ucla.edu/dip	[16]
IntAct	Release 165, updated on Jun 2013	An open-source, open data molecular interaction database populated by data either curated from the literature or from direct data depositions.	Mass spectrometry-based affinity proteomics, and other techniques.	http://www.ebi.ac.uk/intact	[17]
HomoMINT	September 2001 release, updated on 2004	A tool extending experimentally verified protein interactions with its accuracy accessed by experimental interactome, protein networks, and sharing of Gene Ontology annotation.	High throughput approaches based on the yeast two hybrid, TAP TAG methods, and genome-wide approaches.	http://mint.bio.uniroma2.it/HomoMINT	[18]
BioGRID	Version 3.2.99, updated on April 1, 2013	Database containing genetic and physical interaction data from many sources, including several genome/proteome-wide studies, the MIPS database, and BIND.	Luminescence, mass spectrometric methods, two-hybrid, co-fractionation, co-purification, co-crystal structure, fragment complementation assay, and immunoprecipitation.	http://thebiogrid.org/	[19]
