# Supplementary material for: A Systematic In Silico Mining of the Mechanistic Implications and Therapeutic Potentials of Estrogen Receptor (ER)-α in Breast Cancer
Source: PLoS One. 2014 Mar 10;9(3):e91894. doi: 10.1371/journal.pone.0091894 (PMC3948898; doi:10.1371/journal.pone.0091894)
Supplement: Table S2 — Overview of the resources providing experimentally verified MTIs in our study. (PDF) [file pone.0091894.s003.pdf]

Table S2. Overview of the resources providing experimentally verified MTIs in our study.

Programs	Version information	Brief description	Experimental techniques	Web server	References
miRTarBase	Version 4.5	Data were accumulated by manually surveying literature after filtering research articles related to functional studies of miRNAs.	Reporter assays, western blot, or microarray experiments with overexpression or knockdown of miRNAs.	http://mirtarbase.mbc.nctu.edu.tw	[22]
TarBase	Version 6.0	Database hosting the largest collection of manually curated experimentally validated miRNA-gene interactions.	Reporter genes, qPCR, western blotting, microArray, proteomics, sequencing, degradome-Seq, and other (e.g. ELISA, RACE, immunohistochemistry, *etc*.).	http://www.microrna.gr/tarbase	[23]
starBase	Version 1.0	A public platform for comprehensive exploration of miRNA-target interaction maps from CLIP-Seq and degradome sequencing data.	CLIP-Seq (HITS-CLIP, PAR-CLIP) and degradome sequencing (Degradome-Seq, PARE).	http://starbase.sysu.edu.cn	[24]
miRecords	Version 3, updated on April 27, 2013	Resource hosting large, high-quality manually curated miRNA-target interactions with systematic documentation of experimental support.	Most are "low throughput" experiments mRNA-level (target gene, target region, and target site level), protein-level, and others.	http://mirecords.biolead.org	[25]
miRWalk	Version updated on March 15, 2011	Database providing experimentally verified miRNA interactions associated with genes, pathways, organs, transcription factors, cell lines and diseases.	Techniques exploring the involvement of miRNA with genes, proteins, diseases, organs, cell lines, pathways and OMIM disorders.	http://www.umm.uni-heidelberg.de/apps/zmf/mirwalk	[26]
