# Supplementary material for: A Systematic In Silico Mining of the Mechanistic Implications and Therapeutic Potentials of Estrogen Receptor (ER)-α in Breast Cancer
Source: PLoS One. 2014 Mar 10;9(3):e91894. doi: 10.1371/journal.pone.0091894 (PMC3948898; doi:10.1371/journal.pone.0091894)
Supplement: Table S3 — Overview of the three algorithmically different programs providing predicted MTIs in our study. (PDF) [file pone.0091894.s004.pdf]

Table S3. Overview of the three algorithmically different programs providing predicted MTIs in our study.

Programs	Version information	Brief description	Data selection criterion	Web server	References
DIANA-microT-CDS	Version 5.0	The only algorithm available online specifically designed to identify miRNA recognition elements (MREs) both in 3'untranslated region (3'-UTR) and in coding sequences (CDS).	Predictions with strict miTG scores of 0.7 or higher.	http://www.microrna.gr/microT-CDS	[27]
MiRanda	August 2010 release, updated November 1, 2010	A scanning algorithm based on sequence complementarity between mature miRNA and the target site, binding energy of miRNA-target duplex, and evolutionary conservation of the target site sequence and target position in aligned UTRs of homologous genes.	Predictions with ‘‘good’’ mirSVR score of -0.1 or lower.	http://www.microrna.org/microrna	[28]
TargetScan	Version 6.2	The overall scoring of a miRNA target site depends on the level of conservation by searching for the presence of conserved 8mer and 7mer sites that match the seed region of each miRNA.	Predictions with at least one conserved site, appropriate P_{CT} value, and a context+ score of -1.67 or higher.	http://www.targetscan.org	[29]
