# Supplementary material for: A Systematic In Silico Mining of the Mechanistic Implications and Therapeutic Potentials of Estrogen Receptor (ER)-α in Breast Cancer
Source: PLoS One. 2014 Mar 10;9(3):e91894. doi: 10.1371/journal.pone.0091894 (PMC3948898; doi:10.1371/journal.pone.0091894)
Supplement: Table S5 — Reported ER-α-regulating miRNAs. (PDF) [file pone.0091894.s006.pdf]

Table S5. Reported ER-α-regulating miRNAs.

Reported ER-α-regulating miRNAs				
hsa-miR-9	hsa-miR-19a	hsa-miR-20a	hsa-miR-25	hsa-miR-15a
hsa-miR-26b	hsa-miR-27b	hsa-miR-34a	hsa-miR-135a	hsa-miR-24
hsa-miR-219	hsa-miR-450b	hsa-miR-542	hsa-miR-653	hsa-miR-193b
hsa-miR-1234	hsa-miR-125a	hsa-miR-191	hsa-miR-206	hsa-miR-26a
hsa-miR-342	hsa-miR-652	hsa-miR-181a	hsa-miR-221	hsa-miR-375
hsa-miR-18a	hsa-miR-19b	hsa-miR-23a	hsa-miR-140	hsa-miR-425
hsa-miR-27a	hsa-miR-29b	hsa-miR-130b	hsa-miR-181c	hsa-miR-21
hsa-miR-424	hsa-miR-489	hsa-miR-556	hsa-miR-942	hsa-miR-181b
hsa-miR-1290	hsa-miR-145	hsa-miR-196a	hsa-miR-301a	hsa-miR-222
