# Supplementary material for: A Systematic In Silico Mining of the Mechanistic Implications and Therapeutic Potentials of Estrogen Receptor (ER)-α in Breast Cancer
Source: PLoS One. 2014 Mar 10;9(3):e91894. doi: 10.1371/journal.pone.0091894 (PMC3948898; doi:10.1371/journal.pone.0091894)
Supplement: Table S12 — Novel miRNAs predicted to target ER-α from three algorithmically different programs. (PDF) [file pone.0091894.s013.pdf]

**Table S12. Novel miRNAs predicted to target ER-α from three algorithmically different programs.**

| Novel miRNAs | DIANA-microT-CDS | miRanda | TargetScan | | |
|---|---|---|---|---|---|
| | miTG score | mirSVR score | Seed match | Context+ score | $P_{CT}$ |
| hsa-miR-148b | 0.73 | -0.5741 | 7mer-m8 | -0.24 | 0.71 |
| hsa-miR-301b | 0.992 | -0.4375 | 7mer-m8 | -0.20 | 0.83 |
| hsa-miR-302e | 0.962 | -0.3207 | 7mer-m8 | -0.18 | 0.34 |
| hsa-miR-520a-3p | 0.967 | -0.3001 | 7mer-m8 | -0.18 | 0.34 |
| hsa-miR-520b | 0.991 | -0.3127 | 7mer-m8 | -0.20 | 0.34 |
| hsa-miR-520c-3p | 0.991 | -0.295 | 7mer-m8 | -0.20 | 0.34 |
| hsa-miR-520d-3p | 0.979 | -0.3001 | 7mer-m8 | -0.18 | 0.34 |
| hsa-miR-520e | 0.979 | -0.3001 | 7mer-m8 | -0.18 | 0.34 |
| hsa-miR-874 | 0.893 | -0.3836 | 7mer-1A | -0.10 | N/A |
| hsa-miR-1297 | 0.879 | -0.5669 | 7mer-1A | -0.08 | 0.59 |
