# Supplementary material for: A Systematic In Silico Mining of the Mechanistic Implications and Therapeutic Potentials of Estrogen Receptor (ER)-α in Breast Cancer
Source: PLoS One. 2014 Mar 10;9(3):e91894. doi: 10.1371/journal.pone.0091894 (PMC3948898; doi:10.1371/journal.pone.0091894)
Supplement: Table S13 — Performance of different miRNA-target interactions (MTIs) prediction strategies targeting four test datasets. (PDF) [file pone.0091894.s014.pdf]

Table S13. Performance of different miRNA-target interactions (MTIs) prediction strategies targeting four test datasets.

miRNA	Software	Tp	Fp	Tn	Fn	ACC(%)	PPV(%)	FPR(%)
**hsa-miR-30a**	D	9	78	1397	9	94.173	10.345	5.288
	M	14	471	1004	4	68.185	2.887	31.932
	T	3	15	1460	15	97.991	16.667	1.017
	D∩T	2	10	1465	16	98.259	16.667	0.678
	M∩D	8	60	1416	9	95.378	11.765	4.065
	M∩T	3	12	1463	15	98.192	20.000	0.814
	D∪T	10	83	1392	8	93.905	10.753	5.627
	M∪D	14	490	985	4	66.912	2.778	33.220
	M∪T	14	474	1001	4	67.984	2.869	32.136
	(D∩T)∪(M∩T)∪(M∩D)	10	65	1410	8	95.111	13.333	4.407
	(D∪T)∩(M∪T)∩(M∪D)	10	65	1410	8	95.111	13.333	4.407
	D∪M∪T	14	491	984	4	66.845	2.772	33.288
	D∩T∩M	2	8	1467	16	98.392	20.000	0.542
**hsa-miR-1**	D	29	45	1634	11	96.742	39.189	2.680
	M	27	181	1498	13	88.714	12.981	10.780
	T	17	23	1656	23	97.324	42.500	1.370
	D∩T	15	12	1667	25	97.848	55.556	0.715
	M∩D	25	25	1654	15	97.673	50.000	1.489
	M∩T	13	14	1665	27	97.615	48.148	0.834
	D∪T	31	56	1623	9	96.219	35.632	3.335
	M∪D	31	201	1478	9	87.784	13.362	11.971
	M∪T	31	190	1489	9	88.424	14.027	11.316
	(D∩T)∪(M∩T)∪(M∩D)	27	35	1644	13	97.208	43.548	2.085
	(D∪T)∩(M∪T)∩(M∪D)	27	35	1644	13	97.208	43.548	2.085
	D∪M∪T	33	206	1473	7	87.609	13.808	12.269
	D∩T∩M	13	8	1671	27	97.964	61.905	0.476
**hsa-miR-155**	D	24	25	1508	34	96.292	48.980	1.631
	M	34	328	1205	24	77.876	9.392	21.396
	T	24	9	1524	34	97.297	72.727	0.587
	D∩T	19	6	1527	39	97.172	76.000	0.391
	M∩D	22	11	1522	36	97.046	66.667	0.718
	M∩T	21	8	1525	37	97.172	72.414	0.522
	D∪T	29	28	1505	29	96.417	50.877	1.826
	M∪D	36	342	1191	22	77.121	9.524	22.309
	M∪T	37	329	1204	21	78.001	10.109	21.461
	(D∩T)∪(M∩T)∪(M∩D)	28	15	1518	30	97.172	65.116	0.978
	(D∪T)∩(M∪T)∩(M∪D)	28	15	1518	30	97.172	65.116	0.978
	D∪M∪T	37	342	1191	21	77.184	9.763	22.309
	D∩T∩M	16	5	1528	42	97.046	76.190	0.326
**hsa-let-7b**	D	3	102	1356	12	92.261	2.857	6.996
	M	8	510	948	7	64.902	1.544	34.979
	T	0	0	1458	15	98.982	-	0
	D∩T	0	0	1458	15	98.982	-	0
	M∩D	1	90	1368	14	92.940	1.099	6.173

M∩T	0	0	1458	15	98.982	-	0
D∪T	3	102	1356	12	92.261	2.857	6.996
M∪D	3	102	1356	12	92.261	2.857	6.996
M∪T	8	510	948	7	64.902	1.544	34.979
(D∩T)∪(M∩T)∪(M∩D)	1	90	1368	14	92.940	1.099	6.173
(D∪T)∩(M∪T)∩(M∪D)	1	90	1368	14	92.940	1.099	6.173
D∪M∪T	10	522	936	5	64.223	1.880	35.802
D∩T∩M	0	0	1458	15	98.982	-	0
