# Supplementary material for: A Systematic In Silico Mining of the Mechanistic Implications and Therapeutic Potentials of Estrogen Receptor (ER)-α in Breast Cancer
Source: PLoS One. 2014 Mar 10;9(3):e91894. doi: 10.1371/journal.pone.0091894 (PMC3948898; doi:10.1371/journal.pone.0091894)
Supplement: Table S14 — Screening performance of the crystal structure of ER-α LBD (conformation at 0 ps) at different cut-offs. (PDF) [file pone.0091894.s015.pdf]

Table S14. Screening performance of the crystal structure of ER-α LBD (conformation at 0 ps) at different cut-offs.

Cut-off	Tp	Fp	Tn	Fn	ACC(%)	PPV(%)	FPR(%)
10%	7	120	1275	32	89.4	5.512	8.602
20%	11	242	1153	28	81.172	4.348	17.348
30%	15	362	1033	24	73.082	3.979	25.95
40%	16	483	912	23	64.714	3.206	34.624
50%	19	605	790	20	56.416	3.045	43.369
60%	22	722	673	17	48.466	2.957	51.756
70%	29	832	563	10	41.283	3.368	59.642
80%	33	952	443	6	33.194	3.35	68.244
90%	36	1070	325	3	25.174	3.255	76.703
100%	36	1190	205	3	16.806	2.936	85.305
