# Supplementary material for: A Systematic In Silico Mining of the Mechanistic Implications and Therapeutic Potentials of Estrogen Receptor (ER)-α in Breast Cancer
Source: PLoS One. 2014 Mar 10;9(3):e91894. doi: 10.1371/journal.pone.0091894 (PMC3948898; doi:10.1371/journal.pone.0091894)
Supplement: Table S15 — Optimal screening performance of the 26 ER-α LBD conformers at specific cut-offs. (PDF) [file pone.0091894.s016.pdf]

Table S15. Optimal screening performance of the 26 ER-α LBD conformers at specific cut-offs.

Conformer	Cut-off	Tp	Fp	Tn	Fn	ACC(%)	PPV(%)	FPR(%)
0 ps	10%	7	120	1275	32	89.4	5.512	8.602
200 ps	90%	33	1100	295	6	22.873	2.913	78.853
400 ps	100%	34	1156	239	5	19.038	2.857	82.867
600 ps	10%	6	117	1278	33	89.54	4.878	8.387
800 ps	10%	6	123	1272	33	89.121	4.651	8.817
1000 ps	100%	32	1067	328	7	25.105	2.912	76.487
1200 ps	10%	4	134	1261	35	88.215	2.899	9.606
1400 ps	90%	36	1180	215	3	17.503	2.961	84.588
1600 ps	50%	19	660	735	20	52.58	2.798	47.312
1800 ps	100%	30	1186	209	9	16.667	2.467	85.018
2000 ps	100%	34	1241	154	5	13.11	2.667	88.961
2200 ps	100%	37	1249	146	2	12.762	2.877	89.534
2400 ps	100%	33	1301	94	6	8.856	2.474	93.262
2600 ps	90%	35	35	311	4	24.128	3.128	77.706
2800 ps	100%	35	1262	133	4	11.715	2.699	90.466
3000 ps	10%	8	123	1272	31	89.261	6.107	8.817
3200 ps	10%	10	122	1273	29	89.47	7.576	8.746
3400 ps	80%	32	997	398	7	29.986	3.11	71.47
3600 ps	10%	7	125	1270	32	89.052	5.303	8.961
3800 ps	10%	5	128	1267	34	88.703	3.759	9.176
4000 ps	20%	9	241	1154	30	81.102	3.6	17.276
4200 ps	50%	22	582	813	17	58.229	3.642	41.72
4400 ps	10%	5	129	1266	34	88.633	3.731	9.247
4600 ps	100%	37	1266	129	2	11.576	2.84	90.753
4800 ps	10%	7	115	1280	32	89.749	5.738	8.244
5000 ps	100%	32	1067	328	7	25.105	2.912	76.487
