# Supplementary material for: A Systematic In Silico Mining of the Mechanistic Implications and Therapeutic Potentials of Estrogen Receptor (ER)-α in Breast Cancer
Source: PLoS One. 2014 Mar 10;9(3):e91894. doi: 10.1371/journal.pone.0091894 (PMC3948898; doi:10.1371/journal.pone.0091894)
Supplement: Table S16 — Candidate ER-α modulators that might hit all the four valid ER-α LBD conformers. (PDF) [file pone.0091894.s017.pdf]

Table S16. Candidate ER-α modulators that might hit all the four valid ER-α LBD conformers.

Zinc ID	Hit conformations	Grid score	Amber score	Ranking in top 10% candidates
ZINC85549290	0 ps	-20.916264	-36.7675	315/1268
	3000 ps	-37.748447	-59.3239	2/1323
	3200 ps	-35.909554	-30.7325	850/943
	4800 ps	-36.863907	-34.2566	719/1244
ZINC26163116	0 ps	-30.664364	-35.9317	380/1268
	3000 ps	-30.088062	-48.3435	15/1323
	3200 ps	-29.057119	-38.4361	139/943
	4800 ps	-31.636095	-46.4993	44/1244
ZINC33832368	0 ps	-24.765888	-43.0932	85/1268
	3000 ps	-26.962770	-47.6313	19/1323
	3200 ps	-27.686424	-44.1634	29/943
	4800 ps	-26.585775	-42.0767	107/1244
ZINC85596611	0 ps	-78.609940	-41.2648	124/1268
	3000 ps	-62.603687	-46.4521	27/1323
	3200 ps	-64.012138	-42.5078	48/943
	4800 ps	-60.470642	-38.3742	267/1244
ZINC13308494	0 ps	-30.734661	-31.6233	943/1268
	3000 ps	-35.531586	-46.4481	28/1323
	3200 ps	-32.731083	-39.9002	91/943
	4800 ps	-30.803303	-41.0433	133/1244
ZINC85531081	0 ps	-30.244640	-53.8627	14/1268
	3000 ps	-25.324055	-45.7468	31/1323
	3200 ps	-27.855478	-55.763	4/943
	4800 ps	-25.836317	-47.554	37/1244
ZINC04097893	0 ps	-31.941999	-31.5546	957/1268
	3000 ps	-32.880703	-44.8793	37/1323
	3200 ps	-31.691427	-41.8005	60/943
	4800 ps	-29.531178	-42.3606	103/1244
ZINC33830660	0 ps	-38.568840	-54.2314	11/1268
	3000 ps	-25.797998	-44.5275	39/1323
	3200 ps	-27.698788	-51.646	9/943
	4800 ps	-29.986671	-54.9279	10/1244
ZINC85508320	0 ps	-62.757423	-33.2704	690/1268
	3000 ps	-53.691624	-43.7645	47/1323
	3200 ps	-57.899120	-30.2923	932/943
	4800 ps	-53.803299	-37.9686	292/1244
ZINC85541041	0 ps	-32.456211	-32.3677	814/1268
	3000 ps	-25.989622	-42.7629	62/1323
	3200 ps	-28.756353	-42.9781	39/943
	4800 ps	-30.830235	-56.5601	8/1244

ZINC05762254	0 ps	-25.542189	-32.9655	728/1268
	3000 ps	-25.299522	-42.0466	77/1323
	3200 ps	-29.846870	-39.3651	108/943
	4800 ps	-31.021477	-39.2767	216/1244
ZINC85531488	0 ps	-37.391640	-32.1542	859/1268
	3000 ps	-39.281311	-41.1612	93/1323
	3200 ps	-36.163231	-35.954	268/943
	4800 ps	-35.458813	-32.4292	1082/1244
ZINC85543314	0 ps	-39.026505	-37.7976	245/1268
	3000 ps	-27.159281	-41.1529	95/1323
	3200 ps	-30.917027	-36.2361	250/943
	4800 ps	-31.032650	-33.1277	921/1244
ZINC59779670	0 ps	-35.110302	-56.3697	7/1268
	3000 ps	-21.539759	-41.14	96/1323
	3200 ps	-15.376710	-36.973	217/943
	4800 ps	-24.146492	-47.3495	40/1244
ZINC85505858	0 ps	-44.184929	-30.5343	1154/1268
	3000 ps	-52.172024	-40.7513	103/1323
	3200 ps	-47.468136	-36.7614	225/943
	4800 ps	-51.556778	-40.4278	149/1244
ZINC01589535	0 ps	-26.111263	-30.4395	1170/1268
	3000 ps	-27.567383	-40.2846	118/1323
	3200 ps	-28.648808	-37.4628	193/943
	4800 ps	-27.590694	-40.0976	170/1244
ZINC85646189	0 ps	-54.328526	-35.8739	384/1268
	3000 ps	-40.074924	-39.8885	136/1323
	3200 ps	-39.145245	-35.3629	311/943
	4800 ps	-43.350197	-39.6778	190/1244
ZINC38524311	0 ps	-27.494318	-39.4462	182/1268
	3000 ps	-31.221441	-39.8355	141/1323
	3200 ps	-27.663555	-38.1848	154/943
	4800 ps	-28.491741	-36.1332	476/1244
ZINC00968469	0 ps	-25.504211	-39.3965	183/1268
	3000 ps	-28.477156	-39.828	143/1323
	3200 ps	-28.106464	-35.8499	278/943
	4800 ps	-28.413303	-35.7873	518/1244
ZINC34432491	0 ps	-32.094761	-30.8351	1094/1268
	3000 ps	-26.231337	-39.8013	144/1323
	3200 ps	-25.924360	-41.1328	66/943
	4800 ps	-26.715761	-40.5661	143/1244
ZINC14690749	0 ps	-28.933176	-42.3825	94/1268
	3000 ps	-35.093605	-39.6474	148/1323
	3200 ps	-34.616909	-35.8062	281/943
	4800 ps	-32.429695	-33.3453	879/1244

ZINC02173417	0 ps	-41.721073	-34.0217	593/1268
	3000 ps	-36.904362	-39.54	151/1323
	3200 ps	-33.830906	-36.1317	257/943
	4800 ps	-35.530308	-41.1749	131/1244
ZINC01530342	0 ps	-27.655525	-47.2721	35/1268
	3000 ps	-20.928095	-39.5387	152/1323
	3200 ps	-20.355244	-51.5721	10/943
	4800 ps	-22.990967	-45.1549	61/1244
ZINC85543015	0 ps	-31.623184	-40.4298	151/1268
	3000 ps	-31.380383	-39.1581	166/1323
	3200 ps	-30.921295	-30.718	852/943
	4800 ps	-34.745064	-43.5849	80/1244
ZINC85596678	0 ps	-60.114105	-31.5582	956/1268
	3000 ps	-50.065918	-38.9736	172/1323
	3200 ps	-50.403049	-44.8649	25/943
	4800 ps	-52.103363	-37.9438	298/1244
ZINC85508027	0 ps	-67.154480	-39.834	168/1268
	3000 ps	-58.485329	-38.8604	179/1323
	3200 ps	-56.479507	-33.0961	497/943
	4800 ps	-54.012661	-34.3244	709/1244
ZINC59587094	0 ps	-25.352539	-37.7952	246/1268
	3000 ps	-25.987272	-38.604	191/1323
	3200 ps	-25.680960	-32.1423	616/943
	4800 ps	-25.388746	-37.7239	320/1244
ZINC85596608	0 ps	-70.271774	-34.4574	536/1268
	3000 ps	-66.383591	-38.2384	211/1323
	3200 ps	-54.289581	-31.9909	644/943
	4800 ps	-62.649204	-35.7605	523/1244
ZINC14922261	0 ps	-38.101883	-32.1411	862/1268
	3000 ps	-30.160576	-38.1559	218/1323
	3200 ps	-34.206306	-37.7524	175/943
	4800 ps	-34.860847	-39.9338	178/1244
ZINC85543340	0 ps	-40.629795	-44.2453	68/1268
	3000 ps	-29.924133	-38.1289	219/1323
	3200 ps	-26.257351	-31.6635	694/943
	4800 ps	-33.037037	-32.6491	1020/1244
ZINC33831297	0 ps	-35.142540	-44.1294	70/1268
	3000 ps	-24.600582	-37.8395	231/1323
	3200 ps	-27.450941	-38.2184	151/943
	4800 ps	-25.632067	-37.6953	324/1244
ZINC60290133	0 ps	-32.808598	-41.3908	119/1268
	3000 ps	-33.891918	-37.6957	238/1323
	3200 ps	-29.348759	-30.5317	886/943
	4800 ps	-31.110588	-40.0933	171/1244

ZINC60288940	0 ps	-32.399082	-42.3786	95/1268
	3000 ps	-34.336906	-37.6135	243/1323
	3200 ps	-29.360527	-35.7787	285/943
	4800 ps	-31.438684	-38.483	260/1244
ZINC85509819	0 ps	-21.757881	-35.8799	383/1268
	3000 ps	-18.521801	-37.4563	252/1323
	3200 ps	-27.222792	-34.232	396/943
	4800 ps	-27.594492	-36.0698	486/1244
ZINC70455208	0 ps	-37.726547	-48.655	29/1268
	3000 ps	-26.736233	-37.4151	256/1323
	3200 ps	-26.633257	-45.4227	21/943
	4800 ps	-28.243031	-50.6314	24/1244
ZINC85628518	0 ps	-53.317284	-30.1113	1248/1268
	3000 ps	-52.622616	-37.0404	283/1323
	3200 ps	-46.545364	-30.3	931/943
	4800 ps	-52.520248	-34.2365	722/1244
ZINC70454077	0 ps	-21.767212	-104.534	2/1268
	3000 ps	-22.261322	-37.0069	288/1323
	3200 ps	-26.513159	-117.304	1/943
	4800 ps	-27.028753	-38.0919	284/1244
ZINC70454884	0 ps	-37.413967	-31.5246	964/1268
	3000 ps	-31.692980	-36.8343	306/1323
	3200 ps	-33.547371	-34.7597	353/943
	4800 ps	-37.365669	-38.5437	256/1244
ZINC85505959	0 ps	-52.664207	-35.3104	442/1268
	3000 ps	-47.596283	-36.8047	311/1323
	3200 ps	-46.312981	-33.5813	447/943
	4800 ps	-46.290215	-36.4888	431/1244
ZINC67912000	0 ps	-49.495094	-41.3637	120/1268
	3000 ps	-45.099827	-36.7922	316/1323
	3200 ps	-46.705822	-31.3695	734/943
	4800 ps	-44.353832	-31.7961	1232/1244
ZINC59586861	0 ps	-40.878662	-41.9954	105/1268
	3000 ps	-32.972477	-36.6936	327/1323
	3200 ps	-35.978222	-44.1528	30/943
	4800 ps	-36.560490	-41.9626	112/1244
ZINC85543148	0 ps	-32.276039	-34.6433	513/1268
	3000 ps	-25.952730	-36.6113	337/1323
	3200 ps	-28.311329	-40.417	80/943
	4800 ps	-38.413410	-56.7648	6/1244
ZINC70455109	0 ps	-19.690567	-34.4351	539/1268
	3000 ps	-22.487984	-36.1204	388/1323
	3200 ps	-22.133593	-35.5921	295/943
	4800 ps	-21.909857	-37.2117	366/1244

ZINC85550143	0 ps	-37.279373	-40.4634	150/1268
	3000 ps	-31.453947	-36.0168	404/1323
	3200 ps	-29.940079	-33.7148	438/943
	4800 ps	-32.303432	-40.8793	136/1244
ZINC85531491	0 ps	-41.117451	-33.7067	633/1268
	3000 ps	-41.996838	-35.9098	417/1323
	3200 ps	-38.431858	-35.2216	323/943
	4800 ps	-38.418064	-39.3262	211/1244
ZINC85550108	0 ps	-42.731602	-35.1298	467/1268
	3000 ps	-34.134022	-35.7993	429/1323
	3200 ps	-32.823574	-35.6193	294/943
	4800 ps	-33.751900	-37.7932	311/1244
ZINC70454732	0 ps	-33.271236	-30.5423	1153/1268
	3000 ps	-35.182205	-35.4906	460/1323
	3200 ps	-32.523514	-32.3189	594/943
	4800 ps	-35.283543	-34.3131	712/1244
ZINC85629262	0 ps	-47.104279	-36.3179	354/1268
	3000 ps	-46.039494	-35.3271	486/1323
	3200 ps	-46.185917	-36.1617	255/943
	4800 ps	-46.094742	-32.5693	1042/1244
ZINC85531698	0 ps	-27.100735	-32.7525	750/1268
	3000 ps	-24.229435	-35.2739	494/1323
	3200 ps	-26.954580	-37.1312	209/943
	4800 ps	-31.577452	-40.2651	160/1244
ZINC70454731	0 ps	-33.941166	-31.5021	968/1268
	3000 ps	-35.043427	-35.2294	500/1323
	3200 ps	-33.222633	-32.5688	558/943
	4800 ps	-36.501678	-37.0463	382/1244
ZINC06031047	0 ps	-32.953720	-39.3172	185/1268
	3000 ps	-25.936062	-35.1929	503/1323
	3200 ps	-26.208136	-45.9458	18/943
	4800 ps	-30.862852	-32.1915	1134/1244
ZINC85531509	0 ps	-38.959270	-34.1478	571/1268
	3000 ps	-36.539223	-35.1768	506/1323
	3200 ps	-33.778786	-32.4979	571/943
	4800 ps	-34.757084	-35.6084	538/1244
ZINC85625506	0 ps	-62.226669	-34.9465	480/1268
	3000 ps	-56.819420	-34.9987	524/1323
	3200 ps	-63.467964	-35.2909	319/943
	4800 ps	-55.750851	-36.4677	436/1244
ZINC85549939	0 ps	-33.662205	-36.7126	319/1268
	3000 ps	-39.819916	-34.9972	525/1323
	3200 ps	-46.000832	-42.4078	51/943
	4800 ps	-44.950409	-42.7201	97/1244

ZINC85625459	0 ps	-70.748375	-37.5695	260/1268
	3000 ps	-39.819916	-34.8878	544/1323
	3200 ps	-56.444260	-30.4233	908/943
	4800 ps	-54.000160	-41.436	121/1244
ZINC60290089	0 ps	-30.651997	-35.0461	475/1268
	3000 ps	-30.965439	-34.7466	559/1323
	3200 ps	-27.767347	-32.4957	572/943
	4800 ps	-28.707718	-33.1999	903/1244
ZINC85547185	0 ps	-53.076157	-34.7973	492/1268
	3000 ps	-45.914925	-34.3561	605/1323
	3200 ps	-49.834713	-37.9619	163/943
	4800 ps	-51.793228	-47.3806	39/1244
ZINC85626753	0 ps	-54.800777	-30.6035	1142/1268
	3000 ps	-54.070595	-34.0114	652/1323
	3200 ps	-51.094013	-45.4304	20/943
	4800 ps	-54.289661	-37.8012	310/1244
ZINC01597167	0 ps	-28.932493	-34.3875	541/1268
	3000 ps	-29.619520	-33.986	656/1323
	3200 ps	-26.218998	-33.1189	493/943
	4800 ps	-28.816109	-33.6186	822/1244
ZINC02173412	0 ps	-37.510635	-30.2676	1209/1268
	3000 ps	-40.806610	-33.9545	662/1323
	3200 ps	-36.919182	-35.5208	296/943
	4800 ps	-35.407856	-36.4465	441/1244
ZINC85547121	0 ps	-53.401154	-35.958	378/1268
	3000 ps	-48.686684	-33.9292	669/1323
	3200 ps	-50.856110	-33.2164	482/943
	4800 ps	-53.756680	-41.8076	114/1244
ZINC31155872	0 ps	-40.809345	-38.7597	207/1268
	3000 ps	-27.254993	-33.6902	722/1323
	3200 ps	-26.351128	-32.2573	603/943
	4800 ps	-30.254868	-33.1402	918/1244
ZINC85543038	0 ps	-41.191830	-34.0543	586/1268
	3000 ps	-33.916344	-33.5244	746/1323
	3200 ps	-35.453644	-39.7481	96/943
	4800 ps	-34.097710	-41.0308	134/1244
ZINC85529987	0 ps	-33.692898	-42.0854	99/1268
	3000 ps	-24.240669	-33.3661	770/1323
	3200 ps	-34.830589	-62.1763	3/943
	4800 ps	-27.613895	-40.6676	138/1244
ZINC85629196	0 ps	-56.759163	-36.6995	320/1268
	3000 ps	-55.987423	-33.2959	779/1323
	3200 ps	-52.154480	-39.3758	107/943
	4800 ps	-52.328697	-34.3463	706/1244

ZINC03881456	0 ps	-27.403484	-34.5463	523/1268
	3000 ps	-29.046251	-33.2078	797/1323
	3200 ps	-26.392368	-34.4084	378/943
	4800 ps	-27.212862	-31.834	1225/1244
ZINC60290135	0 ps	-32.751862	-31.807	916/1268
	3000 ps	-33.298935	-33.1957	800/1323
	3200 ps	-29.234234	-31.6235	701/943
	4800 ps	-31.268961	-35.6518	534/1244
ZINC33831299	0 ps	-35.865849	-41.9716	108/1268
	3000 ps	-25.983007	-32.9871	832/1323
	3200 ps	-27.165642	-42.6424	43/943
	4800 ps	-24.833826	-31.9533	1180/1244
ZINC85549344	0 ps	-50.992634	-38.3388	218/1268
	3000 ps	-40.544708	-32.7617	871/1323
	3200 ps	-33.600788	-32.6932	534/943
	4800 ps	-41.298553	-37.7411	316/1244
ZINC70455235	0 ps	-45.355942	-36.9011	301/1268
	3000 ps	-40.616505	-32.746	873/1323
	3200 ps	-44.648022	-42.2217	54/943
	4800 ps	-44.082703	-38.1564	280/1244
ZINC85629487	0 ps	-60.502167	-33.7288	630/1268
	3000 ps	-55.259548	-32.7397	875/1323
	3200 ps	-58.186657	-31.2904	751/943
	4800 ps	-56.556496	-34.9303	623/1244
ZINC85628420	0 ps	-61.403790	-35.856	386/1268
	3000 ps	-55.398190	-32.6989	882/1323
	3200 ps	-58.588749	-37.8225	173/943
	4800 ps	-56.936058	-43.3782	86/1244
ZINC85543332	0 ps	-33.802544	-43.4568	79/1268
	3000 ps	-27.243546	-32.301	965/1323
	3200 ps	-30.431742	-38.2533	146/943
	4800 ps	-28.878143	-36.706	415/1244
ZINC70454094	0 ps	-41.846451	-38.9594	199/1268
	3000 ps	-31.574093	-32.0575	1014/1323
	3200 ps	-34.935574	-48.8411	13/943
	4800 ps	-29.589226	-32.3194	1103/1244
ZINC85571219	0 ps	-51.384262	-31.9099	901/1268
	3000 ps	-44.710304	-32.0239	1019/1323
	3200 ps	-39.921925	-34.4024	380/943
	4800 ps	-46.916161	-33.9968	765/1244
ZINC85625432	0 ps	-62.490005	-36.7529	317/1268
	3000 ps	-57.728809	-31.9564	1033/1323
	3200 ps	-59.400017	-37.0564	215/943
	4800 ps	-51.444515	-34.881	630/1244

ZINC70455098	0 ps	-38.394909	-32.2599	839/1268
	3000 ps	-35.071053	-31.5381	1131/1323
	3200 ps	-34.275505	-34.1454	408/943
	4800 ps	-33.286659	-33.9513	774/1244
ZINC85530408	0 ps	-35.148842	-38.6746	209/1268
	3000 ps	-32.726032	-31.287	1192/1323
	3200 ps	-33.822357	-39.8204	94/943
	4800 ps	-34.760006	-37.3779	355/1244
ZINC85543119	0 ps	-53.368698	-47.1583	38/1268
	3000 ps	-34.905506	-31.1555	1224/1323
	3200 ps	-33.986225	-31.3222	744/943
	4800 ps	-46.861900	-45.9804	48/1244
ZINC85596602	0 ps	-68.273453	-31.334	996/1268
	3000 ps	-50.663639	-31.0998	1237/1323
	3200 ps	-64.815483	-36.1789	254/943
	4800 ps	-57.177284	-37.5101	343/1244
